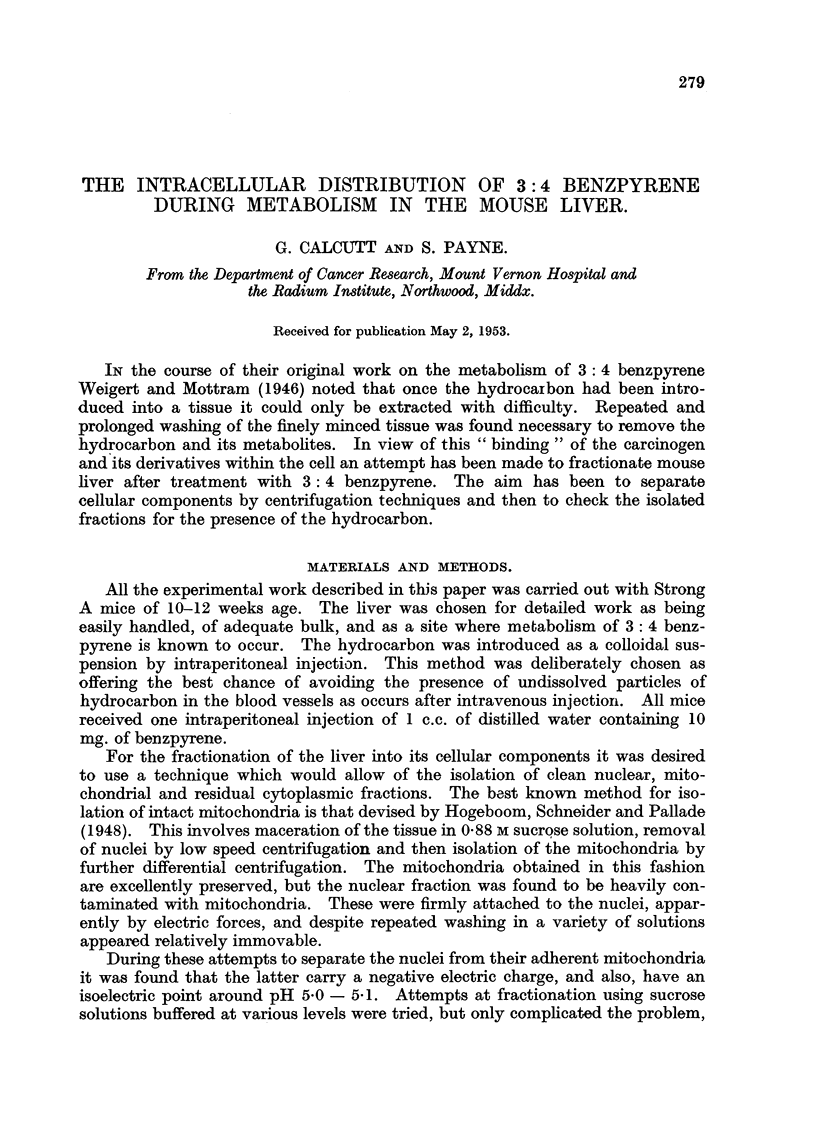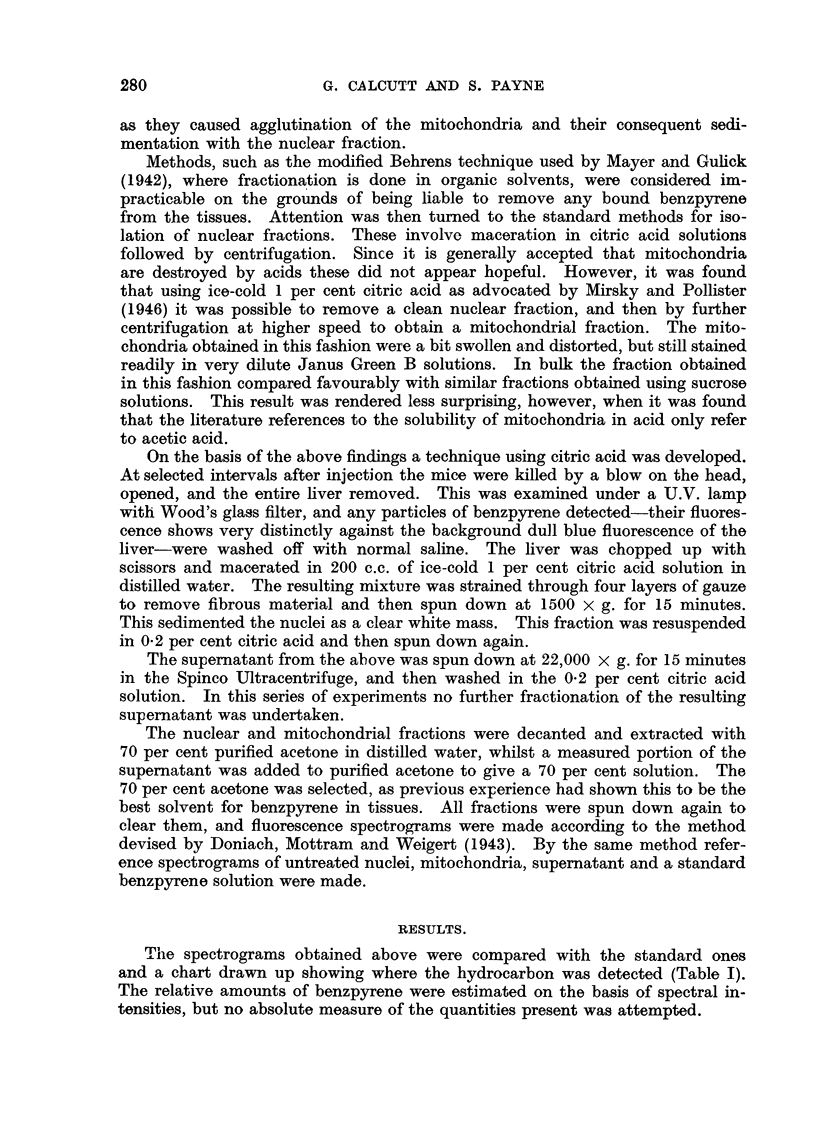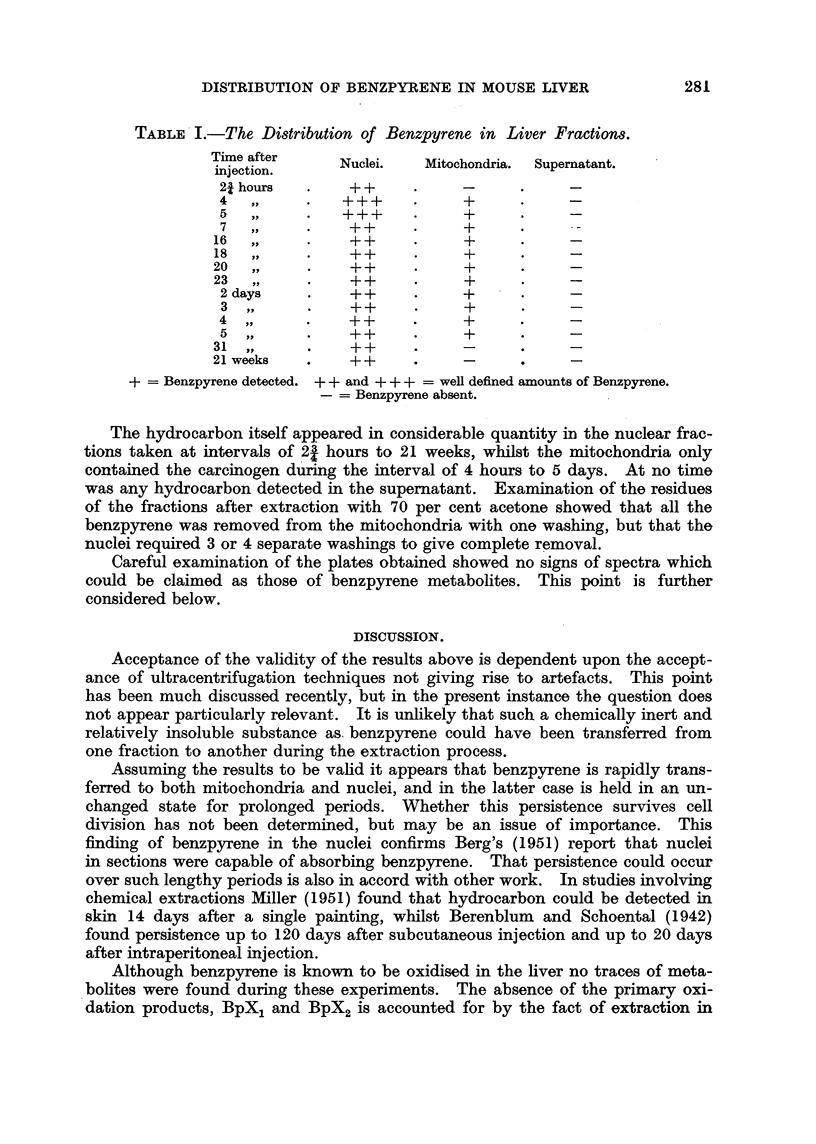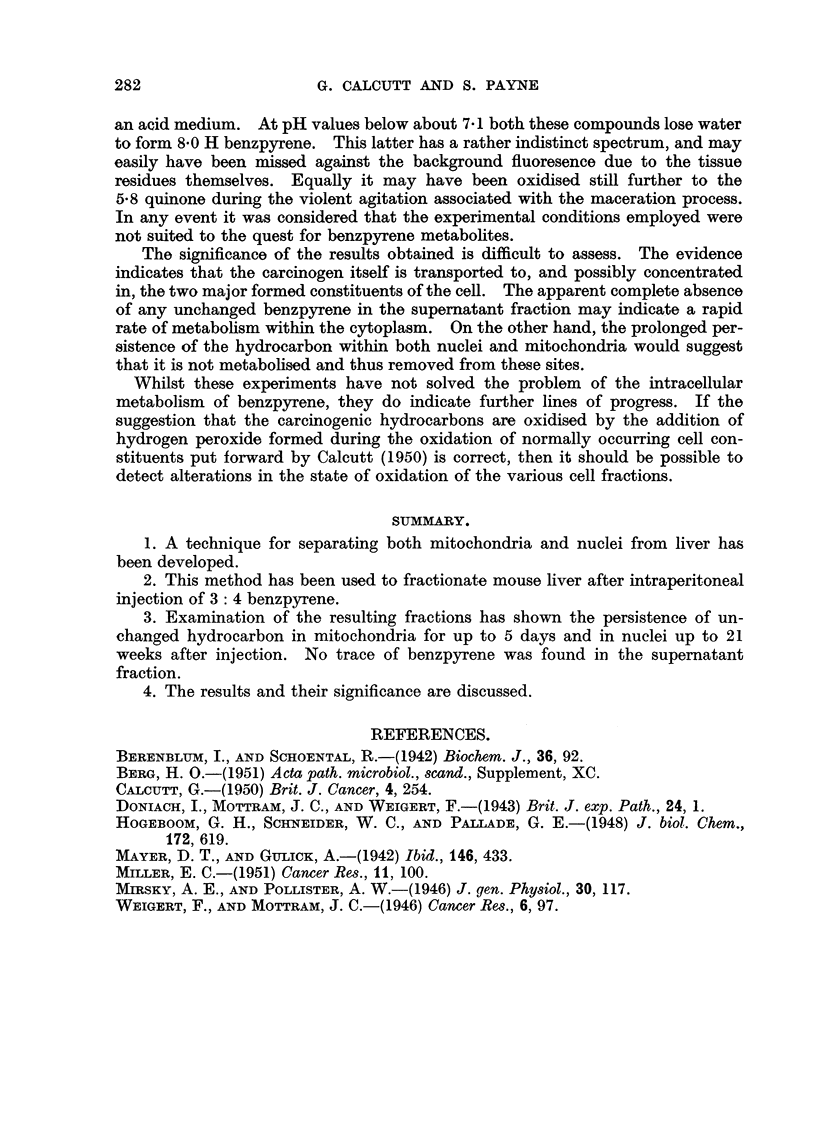# The Intracellular Distribution of 3:4 Benzpyrene during Metabolism in the Mouse Liver

**DOI:** 10.1038/bjc.1953.27

**Published:** 1953-06

**Authors:** G. Calcutt, S. Payne


### THE INTRACELLULAR DISTRIBUTION OF 3:4 BENZPYRENE DURING METABOLISM IN THE MOUSE LIVER.

G. CALCUTT AND S. PAYNE.

*From the Department of Cancer Research, Mount Vernon Hospital and the Radium Institute, Northwood, Middx.*

Received for publication May 2, 1953.

IN the course of their original work on the metabolism of 3 : 4 benzpyrene Weigert and Mottram (1946) noted that once the hydrocarbon had been introduced into a tissue it could only be extracted with difficulty. Repeated and prolonged washing of the finely minced tissue was found necessary to remove the hydrocarbon and its metabolites. In view of this " binding " of the carcinogen and its derivatives within the cell an attempt has been made to fractionate mouse liver after treatment with 3 : 4 benzpyrene. The aim has been to separate cellular components by centrifugation techniques and then to check the isolated fractions for the presence of the hydrocarbon.

### MATERIALS AND METHODS.

All the experimental work described in this paper was carried out with Strong A mice of 10–12 weeks age. The liver was chosen for detailed work as being easily handled, of adequate bulk, and as a site where metabolism of 3 : 4 benzpyrene is known to occur. The hydrocarbon was introduced as a colloidal suspension by intraperitoneal injection. This method was deliberately chosen as offering the best chance of avoiding the presence of undissolved particles of hydrocarbon in the blood vessels as occurs after intravenous injection. All mice received one intraperitoneal injection of 1 c.c. of distilled water containing 10 mg. of benzpyrene.

For the fractionation of the liver into its cellular components it was desired to use a technique which would allow of the isolation of clean nuclear, mitochondrial and residual cytoplasmic fractions. The best known method for isolation of intact mitochondria is that devised by Hogeboom, Schneider and Pallade (1948). This involves maceration of the tissue in 0·88 M sucrose solution, removal of nuclei by low speed centrifugation and then isolation of the mitochondria by further differential centrifugation. The mitochondria obtained in this fashion are excellently preserved, but the nuclear fraction was found to be heavily contaminated with mitochondria. These were firmly attached to the nuclei, apparently by electric forces, and despite repeated washing in a variety of solutions appeared relatively immovable.

During these attempts to separate the nuclei from their adherent mitochondria it was found that the latter carry a negative electric charge, and also, have an isoelectric point around pH 5·0 — 5·1. Attempts at fractionation using sucrose solutions buffered at various levels were tried, but only complicated the problem, as they caused agglutination of the mitochondria and their consequent sedimentation with the nuclear fraction.

Methods, such as the modified Behrens technique used by Mayer and Gulick (1942), where fractionation is done in organic solvents, were considered impracticable on the grounds of being liable to remove any bound benzpyrene from the tissues. Attention was then turned to the standard methods for isolation of nuclear fractions. These involve maceration in citric acid solutions followed by centrifugation. Since it is generally accepted that mitochondria are destroyed by acids these did not appear hopeful. However, it was found that using ice-cold 1 per cent citric acid as advocated by Mirsky and Pollister (1946) it was possible to remove a clean nuclear fraction, and then by further centrifugation at higher speed to obtain a mitochondrial fraction. The mitochondria obtained in this fashion were a bit swollen and distorted, but still stained readily in very dilute Janus Green B solutions. In bulk the fraction obtained in this fashion compared favourably with similar fractions obtained using sucrose solutions. This result was rendered less surprising, however, when it was found that the literature references to the solubility of mitochondria in acid only refer to acetic acid.

On the basis of the above findings a technique using citric acid was developed. At selected intervals after injection the mice were killed by a blow on the head, opened, and the entire liver removed. This was examined under a U.V. lamp with Wood's glass filter, and any particles of benzpyrene detected—their fluorescence shows very distinctly against the background dull blue fluorescence of the liver—were washed off with normal saline. The liver was chopped up with scissors and macerated in 200 c.c. of ice-cold 1 per cent citric acid solution in distilled water. The resulting mixture was strained through four layers of gauze to remove fibrous material and then spun down at 1500 $\times$ g. for 15 minutes. This sedimented the nuclei as a clear white mass. This fraction was resuspended in 0·2 per cent citric acid and then spun down again.

The supernatant from the above was spun down at 22,000 $\times$ g. for 15 minutes in the Spinco Ultracentrifuge, and then washed in the 0·2 per cent citric acid solution. In this series of experiments no further fractionation of the resulting supernatant was undertaken.

The nuclear and mitochondrial fractions were decanted and extracted with 70 per cent purified acetone in distilled water, whilst a measured portion of the supernatant was added to purified acetone to give a 70 per cent solution. The 70 per cent acetone was selected, as previous experience had shown this to be the best solvent for benzpyrene in tissues. All fractions were spun down again to clear them, and fluorescence spectrograms were made according to the method devised by Doniach, Mottram and Weigert (1943). By the same method reference spectrograms of untreated nuclei, mitochondria, supernatant and a standard benzpyrene solution were made.

### RESULTS.

The spectrograms obtained above were compared with the standard ones and a chart drawn up showing where the hydrocarbon was detected (Table I). The relative amounts of benzpyrene were estimated on the basis of spectral intensities, but no absolute measure of the quantities present was attempted.

TABLE I.—*The Distribution of Benzpyrene in Liver Fractions.*

| Time after injection. | Nuclei. | Mitochondria. | Supernatant. |
|---|---|---|---|
| 2¾ hours | ++ | — | — |
| 4 „ | +++ | + | — |
| 5 „ | +++ | + | — |
| 7 „ | ++ | + | — |
| 16 „ | ++ | + | — |
| 18 „ | ++ | + | — |
| 20 „ | ++ | + | — |
| 23 „ | ++ | + | — |
| 2 days | ++ | + | — |
| 3 „ | ++ | + | — |
| 4 „ | ++ | + | — |
| 5 „ | ++ | + | — |
| 31 „ | ++ | — | — |
| 21 weeks | ++ | — | — |

+ = Benzpyrene detected. ++ and +++ = well defined amounts of Benzpyrene.
— = Benzpyrene absent.

The hydrocarbon itself appeared in considerable quantity in the nuclear fractions taken at intervals of 2¾ hours to 21 weeks, whilst the mitochondria only contained the carcinogen during the interval of 4 hours to 5 days. At no time was any hydrocarbon detected in the supernatant. Examination of the residues of the fractions after extraction with 70 per cent acetone showed that all the benzpyrene was removed from the mitochondria with one washing, but that the nuclei required 3 or 4 separate washings to give complete removal.

Careful examination of the plates obtained showed no signs of spectra which could be claimed as those of benzpyrene metabolites. This point is further considered below.

### DISCUSSION.

Acceptance of the validity of the results above is dependent upon the acceptance of ultracentrifugation techniques not giving rise to artefacts. This point has been much discussed recently, but in the present instance the question does not appear particularly relevant. It is unlikely that such a chemically inert and relatively insoluble substance as benzpyrene could have been transferred from one fraction to another during the extraction process.

Assuming the results to be valid it appears that benzpyrene is rapidly transferred to both mitochondria and nuclei, and in the latter case is held in an unchanged state for prolonged periods. Whether this persistence survives cell division has not been determined, but may be an issue of importance. This finding of benzpyrene in the nuclei confirms Berg's (1951) report that nuclei in sections were capable of absorbing benzpyrene. That persistence could occur over such lengthy periods is also in accord with other work. In studies involving chemical extractions Miller (1951) found that hydrocarbon could be detected in skin 14 days after a single painting, whilst Berenblum and Schoental (1942) found persistence up to 120 days after subcutaneous injection and up to 20 days after intraperitoneal injection.

Although benzpyrene is known to be oxidised in the liver no traces of metabolites were found during these experiments. The absence of the primary oxidation products, $BpX_1$ and $BpX_2$ is accounted for by the fact of extraction in an acid medium. At pH values below about 7·1 both these compounds lose water to form 8·0 H benzpyrene. This latter has a rather indistinct spectrum, and may easily have been missed against the background fluoresence due to the tissue residues themselves. Equally it may have been oxidised still further to the 5·8 quinone during the violent agitation associated with the maceration process. In any event it was considered that the experimental conditions employed were not suited to the quest for benzpyrene metabolites.

The significance of the results obtained is difficult to assess. The evidence indicates that the carcinogen itself is transported to, and possibly concentrated in, the two major formed constituents of the cell. The apparent complete absence of any unchanged benzpyrene in the supernatant fraction may indicate a rapid rate of metabolism within the cytoplasm. On the other hand, the prolonged persistence of the hydrocarbon within both nuclei and mitochondria would suggest that it is not metabolised and thus removed from these sites.

Whilst these experiments have not solved the problem of the intracellular metabolism of benzpyrene, they do indicate further lines of progress. If the suggestion that the carcinogenic hydrocarbons are oxidised by the addition of hydrogen peroxide formed during the oxidation of normally occurring cell constituents put forward by Calcutt (1950) is correct, then it should be possible to detect alterations in the state of oxidation of the various cell fractions.

### SUMMARY.

1. A technique for separating both mitochondria and nuclei from liver has been developed.

2. This method has been used to fractionate mouse liver after intraperitoneal injection of 3 : 4 benzpyrene.

3. Examination of the resulting fractions has shown the persistence of unchanged hydrocarbon in mitochondria for up to 5 days and in nuclei up to 21 weeks after injection. No trace of benzpyrene was found in the supernatant fraction.

4. The results and their significance are discussed.

### REFERENCES.

Berenblum, I., and Schoental, R.—(1942) *Biochem. J.*, **36**, 92.
Berg, H. O.—(1951) *Acta path. microbiol., scand.*, Supplement, XC.
Calcutt, G.—(1950) *Brit. J. Cancer*, **4**, 254.
Doniach, I., Mottram, J. C., and Weigert, F.—(1943) *Brit. J. exp. Path.*, **24**, 1.
Hogeboom, G. H., Schneider, W. C., and Pallade, G. E.—(1948) *J. biol. Chem.*, **172**, 619.
Mayer, D. T., and Gulick, A.—(1942) *Ibid.*, **146**, 433.
Miller, E. C.—(1951) *Cancer Res.*, **11**, 100.
Mirsky, A. E., and Pollister, A. W.—(1946) *J. gen. Physiol.*, **30**, 117.
Weigert, F., and Mottram, J. C.—(1946) *Cancer Res.*, **6**, 97.